# Two Classes of Pigments, Carotenoids and C-Phycocyanin, in Spirulina Powder and Their Antioxidant Activities

**DOI:** 10.3390/molecules23082065

**Published:** 2018-08-17

**Authors:** Woo Sung Park, Hye-Jin Kim, Min Li, Dong Hoon Lim, Jungmin Kim, Sang-Soo Kwak, Chang-Min Kang, Mario G. Ferruzzi, Mi-Jeong Ahn

**Affiliations:** 1 College of Pharmacy and Research Institute of Pharmaceutical Sciences, Gyeongsang National University, Jinju 52828, Korea; pws8822@gmail.com (W.S.P.); black200203@gmail.com (H.-J.K.); 2 Plants for Human Health Institute, North Carolina State University, Kannapolis, NC 28081, USA; mli33@ncsu.edu; 3 Department of Information and Statistics and RINS, Gyeongsang National University, Jinju 52828, Korea; dhlim@gnu.ac.kr; 4 Korea Institute of Toxicology, Jinju 52834, Korea; jungmin.kim@kitox.re.kr (J.K.); cmkang@kitox.re.kr (C.-M.K.); 5 Plant Systems Engineering Research Center, Korea Research Institute of Bioscience and Biotechnology, Daejeon 34141, Korea; sskwak@kribb.re.kr

**Keywords:** *Arthrospira platensis*, carotenoids, natural pigments, spirulina powder, C-phycocyanin, antioxidant activity

## Abstract

*Arthrospira platensis* is the widely available source of spirulina that contains distinctive natural pigments, including carotenoids and C-phycocyanin (C-PC). In this study, the major carotenoid and C-PC contents were determined in seven commercially available spirulina powder products and laboratory-prepared *A. platensis* trichomes (AP-1) by an LC-DAD method and UV-Visible spectrometry, respectively. The correlation of these two pigment content levels with Hunter color coordinates and antioxidant activity was also evaluated. The L* value failed to show a significant correlation with pigment content, but a positive correlation was observed between a* values and the contents of total carotenoid and C-PC. As b* values decreased, the chlorophyll a and C-PC contents increased. AP-1 exhibited the highest content of total carotenoids, chlorophyll a and C-PC, and antioxidant activities among the samples. This observation could be related to degradation of these pigments during the mass production process. The carotenoid profiles suggested that the commercial spirulina powders originated from two different sources, *A. platensis* and *A. maxima*. Total carotenoid and C-PC content exhibited positive significant correlations with antioxidant activities measured by 1,1-diphenyl-2-picrylhydrazyl (DPPH) and 2,2′-azino-bis(3-ethylbenzothiazoline-6-sulphonic acid) (ABTS) assays. These results provide a strong scientific foundation for the establishment of standards for the commercial distribution of quality spirulina products.

## 1. Introduction

Most commercial spirulina powder is composed of dried bodies of *Arthrospira* (*Spirulina*) *platensisis* or *A. maxima* [1]. These species, also known as spirulina, are Gram-negative, nontoxic species of cyanobacteria that are widely used as food supplements or natural additives. Spirulina is regarded as an ideal food and quality drug resource due to its high content of protein, lipid, vitamins, minerals, chlorophyll, β-carotene, and polysaccharides. With two distinctive natural colors, carotenoids and C-phycocyanin [2,3], spirulina use has aligned with consumer awareness regarding the importance of natural color agents for their nutritional, pharmacological and health-related benefits. As a result, the number of applications of natural pigments and spirulina as a source of these pigments is increasing, especially in the food and cosmetic industries [4].

Carotenoids are a class of natural lipid-soluble pigments that are responsible for the red, yellow, and orange colors found in various plants and microorganisms. They function primarily as photosynthesis aids and are used in the photoprotection process. Humans and other animals are unable to synthesize carotenoids and acquire them through alimentation. Carotenoids are used in food and feed as colorants and flavorings, and in nutritional supplements as a source of provitamin A [1]. The health benefits of carotenoids to humans and animals are becoming increasingly apparent. For example, there is evidence that these pigments may protect humans from serious disorders associated with oxidative and inflammatory stress including skin degeneration and aging, cardiovascular disease, certain types of cancer, and age-related diseases of the eye, such as macular degeneration or cataracts [5,6,7].

C-Phycocyanin (C-PC) is a hydrophilic and intense blue-colored biliprotein found in blue green algae. C-PC comprises a protein and the chromophore phycocyanobilin. The protein moiety consists of α and β subunits with molecular weights near 18,000 and 20,000 Da, respectively. This colorant is highly stable in the pH range of 5–8 and exhibits a strong red fluorescence when present in its native form [4]. The biological activities of C-PC are wide-ranging and include antioxidant, antimutative, antiviral and antitumor properties. C-PC has also been shown to stimulate the immune system and exhibit hepatoprotective, antiplatelet, and neuroprotective activities [8,9,10]. It is believed that the various health benefits of spirulina are derived from oxidative stress reduction [11,12], and β-carotene and C-PC are important contributors to this effect [13].

These pigments can be easily degraded during the mass production process using sunlight or hot-air dry. In order to provide improved characterization of available spirulina ingredients to manufacturers and scientistz, the carotenoid and C-PC content levels were determined for seven commercially available spirulina powder products and for freeze-dried *A. platensis* trichomes cultured in our laboratory which could give best condition for retaining these pigments. Additionally, the correlation of pigment levels with the Hunter Lab color parameters was assessed as was the relationship of the pigments to antioxidant activity as a method to better understand predictive quality factors for these ingredients.

## 2. Results

### 2.1. Colorimetric Evaluation of Spirulina Powder

Because carotenoids and C-PC are pigments, a colorimetric evaluation was performed on the spirulina powders obtained from *Athrospira platensis* cultivated in our laboratory (AP-1) or purchased from on-line and local stores (C1–C7). All of the powders were dark green and differed slightly from each other in the Hunter color coordinates of brightness (L*), redness (a*) and yellowness (b*).

The highest L* and b* values were obtained in C6, with C7 displaying the second highest L* value (Table 1). All samples showed negative a* values due to the green color, and the mean b* values were between 6.3 and 13.3.

### 2.2. Carotenoid and C-PC Content in Spirulina Powder

**Figure 1:**
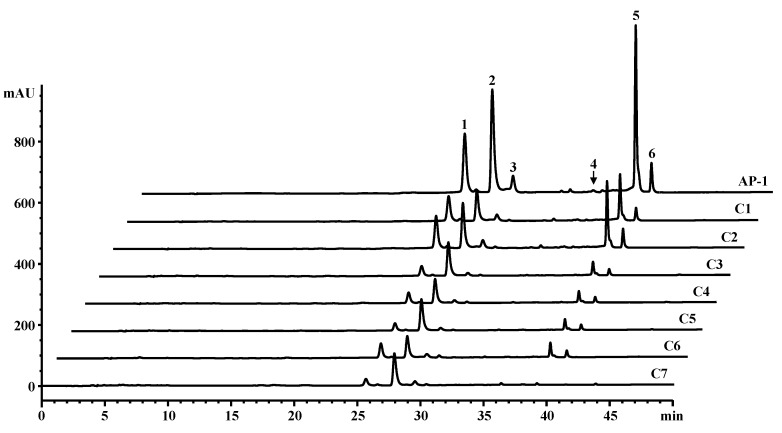
LC chromatogram of carotenoid extract from spirulina samples (450 nm). Peaks 1–6 correspond to all-*trans*-zeaxanthin, chlorophyll a, diatoxanthin, 13-*cis*-β-carotene, all-*trans*-β-carotene and 9-*cis*- β-carotene, respectively.

Calibration curves were constructed by analyzing a mixture containing five carotenoids at various concentration levels and plotting peak area against the concentration of each reference standard (Table 2). The curves showed good linearity, and the correlation coefficients were between 0.997 and 0.999 for all of the compounds over the concentration ranges of quantification. The recovery of four carotenoids excepting diatoxanthin was assessed by spiking samples with high and low concentrations of each reference compound, 1000 and 30 ng, respectively. Spiking with 19.2 and 2.8 ng was done for diatoxanthin. The average recoveries were between 85.6% and 107.4% (*n* = 3). The limits of detection (LOD) were determined by serial dilution based on a signal-to-noise (S/N) ratio of 3:1 (Table 2). The peak purity was determined by the photodiode array detector and the corresponding computer software that confirmed the singularity of each peak. In addition, the absorption spectrum of each peak was compared with the characteristics of each standard compound. All-*trans*-β-carotene, all-*trans*-zeaxanthin, 9-*cis*-β-carotene and diatoxanthin were found to be the major carotenoids present in spirulina (Figure 1 and Table 1). 13-*cis*-β-Carotene was also detected. The content of all-*trans*-β-carotene was highest among the four major carotenoids in AP-1, C1, C2 and C5 while that of all-*trans*-zeaxanthin was highest in the remaining samples. AP-1, C1 and C2 contained more than 1.6 mg/g dry weight of total carotenoids while the other samples contained less than 0.8 mg/g total carotenoid content. AP-1 showed the highest total carotenoid content of 4.43 ± 0.03 mg/g.

The green photosynthetic pigment chlorophyll a which is essential for photosynthesis in cyanobacteria as primary electron donor was also determined at the same LC conditions used for carotenoid analysis with a different wavelength. AP-1 showed the highest level of chlorophyll a, 10.8 ± 1.1 mg/g, while the mean values in the other samples were between 2.6–4.7 mg/g (Table 1).

C-PC, a major biliprotein of spirulina, was extracted by grinding the sample powder with sea sand and sonication at 4 °C. The extraction efficiency observed at pH 7 was higher than at pH 4 and 10 (data not shown). AP-1 showed the greatest amount of C-PC, 251.2 ± 11.2 mg/g, and the mean values in the commercial spirulina samples were between 94.9–153.3 mg/g (Table 1).

Among the three major pigments in spirulina, the content of C-PC was highest with a value of 10–25% (*w*/*w*). The average percentages of chlorophyll a and carotenoids were 0.26–1.1% and 0.03–0.38%, respectively. The total carotenoid content varied by up to eight-fold among the commercial samples, while the content variations of chlorophyll a and C-PC were 1.8 and 1.6-fold, respectively.

### 2.3. Antioxidant Activity of Carotenoid and C-PC Extracts

Carotenoids, phycocyanins and spirulina are known to have potent antioxidant activity. The 1,1-diphenyl-2-picrylhydrazyl (DPPH) and 2,2′-azino-bis(3-ethylbenzothiazoline-6-sulphonic acid) (ABTS) assay methods were applied to determine the antioxidant activity of the carotenoid and C-PC extracts obtained from our spirulina samples. The C-PC extract showed higher antioxidant activities than the carotenoid extract in the DPPH and ABTS assays (Table 3). The carotenoid extract of AP-1 with the highest content of total carotenoids and chlorophyll a exhibited the highest DPPH and ABTS radical scavenging activities among the tested carotenoid extracts. AP-1 with the highest content of C-PC also showed the highest antioxidant activities among the C-PC extract samples. DPPH and ABTS assays displayed slightly different antioxidant activity pattern in carotenoid and C-PC extracts, respectively.

The total phenol and flavonoid contents were evaluated to investigate the effect of phenolic and flavonoid compounds on the antioxidant activities of these extracts. The results showed that the mean value of total phenol content in the carotenoid extract was in the range of 1.3–6.4 μmol gallic acid equivalents (GAE)/g dry weight, which was much lower than that of 47.0–82.1 μmol GAE/g observed in the C-PC extract (Table 3). Additionally, significant negative correlation were observed between total phenolic content in the carotenoid extract with the content of carotenoids, chlorophyll a and flavonoids, and DPPH radical scavenging activity of the extract (Table 4). Meanwhile, the mean flavonoid content in the carotenoid extract was in the range of 12.9–26.6 quercetin equivalents (QE)/g and displayed significant positive correlations with carotenoid and chlorophyll a contents and DPPH radical scavenging activity of the extract. Flavonoids were not detected in the C-PC extract. Total phenol content of the C-PC extract exhibited significant positive correlations with the content level of three pigments and antioxidant activities of the extract.

## 3. Discussion

Spirulina has been used as an additive in a variety of health foods and animal feeds, and has been produced commercially for the last 30 years for these purposes. Commercially grown spirulina is normally produced in large outdoor ponds under controlled conditions or harvested directly from lakes [1]. It contains substantial amount of two distinctive pigmented antioxidants: the yellow-to-red carotenoids and blue C-phycocyanin (C-PC). In this study, the major carotenoid and C-PC content levels were determined using an LC-DAD method and a UV spectrometer, respectively, for seven commercially available spirulina powder products (C1–C7) with freeze-dried *A. platensis* trichomes cultured in our laboratory (AP-1). The correlation of the content level of these two pigments with a visible parameter and antioxidant activity was also evaluated.

The L* value failed to show a significant correlation with pigment content or antioxidant activities (Table 4). As the a* values (redness) increased, the total carotenoid and C-PC content increased. The redness value displayed a non-significant correlation with chlorophyll a content. As the b* values (+yellow −blue) decreased, the chlorophyll a and C-PC content of the extracts increased. Although dark green color of chlorophylls is dominant in the spirulina powders, this result suggests that the redness and blueness would reflect the content of carotenoids and C-PC.

The content of all-*trans*-β-carotene was highest among the four major carotenoid ingredients of the spirulina powder samples in AP-1, C1, C2 and C5 while that of all-*trans*-zeaxanthin was highest in the other samples. The other four samples showed lower a* values than the formers. Spirulina are multicellular and filamentous blue-green microalgae belonging to the two separate genera—*Spirulina* and *Arthrospira*—which consist of approximately 15 species. Of these, *Arthrospira platensis* is the most common and widely available source of spirulina and most of the published research and public health decisions refer to this specific species [14]. Therefore, *A. plantensis* was chosen and cultivated in this study as a control. However, two *Arthrospira* species, *A. platensis* and *A. maxima*, have been used as a food source, dietary supplement, and feed supplement [1]. It has been reported that the content of zeaxanthin was higher than that of β-carotene in a dried *A. maxima* sample [15]. Therefore, the difference in carotenoid profiles between the two groups might result from the fact that the group including AP-1 belonged to *A. platensis* and the other group belonged to *A. maxima*. Contrary to previous findings, β-cryptoxanthin was not detected in the spirulina powder used in this study [2,15]. Instead of β-cryptoxanthin, diatoxanthin was detected along with 9-*cis*-β-carotene and 13-*cis*-β-carotene (Figure 1) [1,14].

There were substantial differences in carotenoid, chlorophyll a and C-PC content between the lyophilized sample AP-1 and commercially available spirulina powders (C1–C7). AP-1 was cultivated and freshly prepared in our laboratory. The total carotenoid, chlorophyll a and C-PC contents observed in AP-1 were the highest seen among the eight samples. AP-1 also exhibited the highest antioxidant activities among the samples. The overall lower amounts of carotenoid, chlorophyll a and C-PC contents found in commercial spirulina powder compared to AP-1 could stem from pigment degradation during the mass production process, as the natural pigments are sensitive to light, heat and oxygen. It has been reported that the carotenoid composition differences of dried commercial spirulina preparations are most likely due to their thermolability [15]. While reportedly stable over a pH range of 5–7.5 at 9 ± 1 °C, thermal instability of C-PC obtained from *A. platensis* above 40 °C has been observed [16]. Chlorophyll degradation is known to occur during the roasting process [17]. A solar or hot-air based drying process is utilized in the mass production of most commercial spirulina powders, which could decrease the pigment content of the powder. Additional food processing techniques and storage conditions can have a similar effect. The antioxidant potential of *A. platensis* powder was easily degraded after exposure of the biomass to heat and light [18].

Total carotenoid content showed high correlation coefficients of 0.92 and 0.86 to chlorophyll a and C-PC content, respectively. The total carotenoid content also exhibited greater positive correlations with antioxidant activities than the total phenol or flavonoid contents of the carotenoid extract did. These strong correlations suggest that the major antioxidant compounds in the carotenoid extract are carotenoids, not phenols or flavonoids, although phenols and flavonoids are also known antioxidants, and the flavonoid content level contributed in portion to the antioxidant activity of the samples with low content of total carotenoids. Chlorophyll a and C-PC contents also showed significant positive correlations, respectively, with the other two pigment contents and antioxidant activities. Different from the carotenoid extract, the total phenol content in C-PC extract showed similar positive correlations with antioxidant activities of this extract compared to the C-PC content in C-PC extract. This result suggests that the phenol compounds in the C-PC extract also contributed to the antioxidant activity in portion, although C-PC is the highest abundant and major antioxidant ingredient of this extract.

The antioxidant activities against DPPH and ABTS radicals displayed overall significant positive correlations to each other in both carotenoid and C-PC extracts. While in the carotenoid extract DPPH assay exhibited greater correlations with the lipophilic total carotenoid, chlorophyll a and flavonoid contents than ABTS assay, in the C-PC extract ABTS assay showed higher positive correlations with the hydrophilic C-PC and total phenolic contents than DPPH assay. This result is well consistent with the previous report that the high-pigmented and hydrophilic antioxidants are better reflected by ABTS assay than DPPH assay [19].

This study revealed that the color attribute of spirulina powder, redness and blueness, would be a factor to assume the lipophilic carotenoid and hydrophilic C-PC contents, and the powder with high level of carotenoid content has also high possibility to show high levels of chlorophyll a and C-PC contents with high antioxidant activity. In addition, the carotenoid profile can be utilized as a parameter for the discrimination of botanical source. These results provide a scientific foundation for proper identification and establishment of standards for distribution of quality commercial spirulina products. Future studies will evaluate the stability of these natural pigments in spirulina powder during storage under various conditions.

## 4. Materials and Methods

### 4.1. Sample Preparation

*Athrospira platensis* (Nordstedt) Gomont (P_PS_00001194) was provided by the Korea Institute of Ocean Science and Technology (Geoje, Korea). Cultivation was conducted for a period of 10 days in 500 mL baffled flasks containing 300 mL of Zarrouk’s medium (Sigma, St. Louis, MO, USA) with stirring at 120 rpm at 25 °C under 130 μmol/m^2^ s illumination. Cells were harvested by centrifugation (Vision Scientific Co., Ltd., VS-24SMTi, Daejeon, Korea) at 3000× *g* for 5 min at 4 °C and freeze-dried (ilShin BioBase Co., Ltd., TFD 5503, Dongducheonsi, Korea, –40 °C). The lyophilized sample (AP) was stored at –80 °C until analysis was completed. The seven commercially available spirulina powder samples (C1–C7) were purchased from various online and in-store sources from November to December in 2017. The cultivation regions of these products included Australia (C7, Ferngrove Pharmaceuticals Pty. Ltd., Chester Hill, Australia), China (C4, Jeoungwoodang, Seoul, Korea), India (C6, Sri Dhanalakshmi Industries, Tamil Nadu, India), New Zealand (C5, New Zealand Nutritionals Ltd., Christchurch, New Zealand) and USA (C1–C3, from three vendors, respectively, of GNM LIFE Ltd., Seoul, Korea; Noksibchocare Industry, Seoul, Korea; Nutraceuticals International Group LLC, Elmwood Park, NJ, USA).

### 4.2. Colorimetric Evaluation

Color attributes of samples were measured with a colorimeter (Konica Minolta CR-400, Osaka, Japan). Prior to testing, the colorimeter was calibrated using a Minolta standard white reflector plate. Color measurements were taken between three and five times per sample, depending on the portion of each powder. The data were presented as L* (lightness), a* (redness), and b* (yellowness) values according to the Hunter color system.

### 4.3. Carotenoid Analysis

All extraction procedures were performed under subdued light to avoid pigment degradation. Two hundred and fifty milligrams of the lyophilized samples were homogenized using a pestle in a prechilled mortar with 1 mL of acetone (stabilized with 0.01% butylated hydroxytoluene, BHT), sea sand, Na_2_SO_4_ and NaHCO_3_. The solution was transferred to a 10 mL conical tube and sonicated three times for 10 min. The extract was centrifuged at 5700× *g* at 4 °C for 10 min (5430R, Eppendorf, Hamburg, Germany), and 5 mL of the supernatant was dried under a stream of N_2_ gas and dissolved in 500 μL of a CH_2_Cl_2_ and acetone mixture (1:1, *v*/*v*). This sample solution was filtered through a 0.45 μm membrane filter (polytetrafluoroethylene (PTFE), 13 mm, 0.45 μm, Whatman, Maidstone, UK) prior to LC analysis.

Carotenoid analysis was conducted according to our previously reported method [20] using an Agilent 1260 HPLC system (Hewlett-Packard, Waldbronn, Germany). Chlorophyll a was also quantified using the same LC conditions, with UV detection set at 430 nm. Under these conditions, standard compound peaks eluted at the following *t*_R_ (min): 25.0 for all-*trans*-zeaxanthin, 27.2 for chlorophyll a, 28.9 for diatoxanthin, 36.3 for 13-*cis*-β-carotene, 39.0 for all-*trans*-β-carotene and 40.3 for 9-*cis*-β-carotene. Methanol, water and methyl-*tert*-butyl ether used in the HPLC system were all of HPLC grade, and all other chemicals were extra grade. A stock solution of diatoxanthin (0.691 mg/L) was purchased from DHI (Hoersholm, Denmark), and the other carotenoids and chlorophyll a were purchased from CaroteNature GmBH (Lupsingen, Switzerland) and Sigma, respectively.

### 4.4. C-PC Analysis

C-PC was quantified by UV-Visible electronic absorption using an external calibration method [4]. Briefly, one hundred milligrams of the lyophilized samples were ground with seasand and extracted by four times sonication for 30 min each with 30 mL of phosphate buffer saline (PBS) (pH 7.0) buffer solution. The extract was centrifuged at 20,000× *g* at 4 °C for 6 h and the supernatant was filtered through a 0.45 μm membrane filter (Whatman, PTFE, 13 mm). Two hundred microliters of each extract was transferred to a 96 well plate, where a microplate reader (BioTek, Synergy H1, Winooski, VT, USA) was used to detect the absorbance at 620 nm. Working calibration solutions in the range of 0.5–5 mg/mL were prepared by diluting the stock solution of C-phycocyanin (Sigma, St. Louis, MO, USA) with PBS buffer. All of the procedures were performed under subdued light to avoid pigment degradation as described above.

### 4.5. Total Phenol and Total Flavonoid Contents

The total phenol content of the extract was determined spectrophotometrically according to our previously described method [21], which is a slightly modified form of the Folin-Ciocalteu colorimetric method [22]. One hundred microliters of the extract was mixed with 500 μL of Folin-Ciocalteu solution (Merck, Darmstadt, Germany) and 400 μL of a 200 mM sodium carbonate solution. After mixing, the solution was centrifuged at 3000× g for 5 min. Two hundred microliters of the upper layer were transferred to a 96-well plate and the absorbance was measured at 765 nm with a microplate reader (BioTek, Synergy H1). Using a gallic acid (Sigma, Hong Kong, China) calibration standard, the results were expressed as μmol gallic acid equivalents per g (μmol GAE/g).

The total flavonoid content was determined using a diethylene glycol colorimetric method [22] using quercetin as the standard. Sample extracts (20 μL) were added to 170 μL of 90% diethylene glycol and 10 μL of a 4 M NaOH solution. The absorbance was measured at 420 nm after 10 min. The results were expressed as μmol quercetin (Sigma-Aldrich, Munich, Germany) equivalents per gram (μmol QE/g).

### 4.6. Antioxidant Activity Test with DPPH Radical

The scavenging activities of the samples on 1,1-diphenyl-2-picrylhydrazyl (DPPH) were measured using a slightly modified form of our previously reported method [21]. The radical scavenging activity was expressed as Trolox (Sigma) equivalents per gram (μmol TE/g).

### 4.7. Antioxidant Activity Test with ABTS Radical

The ABTS radical was generated using a previously reported method [23]. Each extract (20 μL) was reacted with 180 μL of the ABTS^•+^ solution at room temperature, and the absorbance was measured at 734 nm after 10 min. The antioxidant activity of each sample was expressed as Trolox (Sigma) equivalents per gram (μmol TE/g).

### 4.8. Statistical Analysis

All of the contents and the antioxidant activities are expressed as the means ± standard deviations (SD) of triplicate determinations. The differences among samples were statistically evaluated via one-way analysis of variance (ANOVA). The values were evaluated at the 5% significance level using two-sided tests. Pearson’s correlation coefficients were obtained using IBM SPSS Statistics 24.0 software (Armonk, NY, USA).

## Figures and Tables

**Table 1:** Hunter values and pigment contents in spirulina samples.

Items	Samples							
	AP-1	C1	C2	C3	C4	C5	C6	C7
L*	19.2 ± 0.7 ^c^	18.8 ± 0.5 ^c^	16.9 ± 0.9 ^d^	17.4 ± 0.9 ^d^	16.3 ± 0.1 ^d^	14.1 ± 0.6 ^e^	28.0 ± 1.2 ^a^	24.4 ± 0.1 ^b^
a*	−7.4 ± 0.1 ^b^	−7.2 ± 0.1 ^b^	−6.8 ± 0.2 ^a^	−9.0 ± 0.1 ^d^	−8.1 ± 0.3 ^c^	−7.5 ± 0.2 ^b^	−9.5 ± 0.0 ^e^	−10.0 ± 0.0 ^f^
b*	6.9 ± 0.1 ^d^	8.4 ± 0.1 ^b^	7.0 ± 0.2 ^d^	8.7 ± 0.4 ^b^	8.7 ± 0.4 ^b^	6.3 ± 0.1 ^e^	13.3 ± 0.3 ^a^	8.0 ± 0.1 ^c^
All-*trans*-zeaxanthin	1.27 ± 0.12 ^a^	0.54 ± 0.08 ^b^	0.69 ± 0.11 ^b^	0.13 ± 0.02 ^e^	0.22 ± 0.03 ^d^	0.09 ± 0.02 ^f^	0.31 ± 0.01 ^c^	0.12 ± 0.01 ^e^
Diatoxanthin	0.26 ± 0.04 ^a^	0.14 ± 0.03 ^b^	0.17 ± 0.03 ^b^	0.05 ± 0.01 ^d^	0.06 ± 0.00 ^d^	0.05 ± 0.01 ^d^	0.08 ± 0.01 ^c^	0.09 ± 0.01 ^c^
13-*cis*-β-Carotene	0.06 ± 0.01 ^a^	0.02 ± 0.00 ^c^	0.04 ± 0.00 ^b^	0.00 ± 0.00 ^d^	0.01 ± 0.00 ^e^	0.01 ± 0.00 ^d^	0.02 ± 0.00 ^c^	0.01 ± 0.00 ^e^
All-*trans*-β-carotene	2.30 ± 0.05 ^a^	0.72 ± 0.03 ^b^	0.92 ± 0.17 ^b^	0.08 ± 0.01 ^d^	0.16 ± 0.02 ^c^	0.16 ± 0.02 ^c^	0.19 ± 0.02 ^c^	0.02 ± 0.00 ^e^
9-*cis*-β-Carotene	0.38 ± 0.05 ^a^	0.17 ± 0.02 ^c^	0.25 ± 0.03 ^b^	0.05 ± 0.01 ^e^	0.07 ± 0.01 ^e^	0.06 ± 0.01 ^e^	0.09 ± 0.01 ^d^	0.00 ± 0.00 ^f^
Other carotenoids	0.17 ± 0.01 ^a^	0.12 ± 0.01 ^b^	0.16 ± 0.00 ^a^	0.06 ± 0.00 ^d^	0.07 ± 0.01 ^c^	0.05 ± 0.00 ^e^	0.08 ± 0.00 ^c^	0.05 ± 0.01 ^e^
Total carotenoids	4.43 ± 0.03 ^a^	1.70 ± 0.15 ^c^	2.23 ± 0.35 ^b^	0.38 ± 0.03 ^f^	0.58 ± 0.05 ^e^	0.41 ± 0.05 ^f^	0.76 ± 0.03 ^d^	0.28 ± 0.02 ^g^
Chlorophyll a	10.8 ± 1.1 ^a^	3.4 ± 0.3 ^c^	4.7 ± 0.6 ^b^	3.5 ± 0.6 ^c^	2.7 ± 0.2 ^d^	3.3 ± 0.4 ^c^	2.6 ± 0.0 ^d^	3.6 ± 0.5 ^c^
C-Phycocyanin	251.2 ± 11.2 ^a^	100.2 ± 1.1 ^d^	153.3 ± 2.3 ^b^	106.5 ± 1.5 ^c^	113.4 ± 9.1 ^c^	144.8 ± 4.1 ^b^	94.9 ± 6.3 ^d^	108.6 ± 0.7 ^c^

L*, lightness; a*, +red −green; b*, +yellow −blue (CR-400, Minolta). Data are expressed as the mean and SD (the standard deviation value) of three independent experiments. The mean value of pigment contents is the average value of content for dry weight, mg/g). Different letters in the same row mean significantly different (*p* < 0.05).

**Table 2:** Linear ranges and correlation coefficients of calibration curves.

Compounds	Range (µg/mL)	Slope (*a*) ^a^	Intercept (*b*) ^b^	Regression (*r^2^*)	LOD (ng)	LOQ (ng)
All-*trans*-zeaxanthin (1)	0.40–25.0	132.4	14.2	0.9991	0.3	1.0
Chlorophyll a (2)	2.00–250	27.0	−55.6	0.9993	0.7	2.0
Diatoxanthin (3)	0.14–0.69	141.4	0.80	0.9990	0.3	1.0
13-*cis*-β-Carotene (4)	0.08–12.5	128.5	15.0	0.9996	0.3	1.0
All-*trans*-β-carotene (5)	0.14–100	122.6	4.10	0.9997	0.3	1.0
9-*cis*-β-Carotene (6)	0.08–12.5	124.4	6.00	0.9968	0.3	1.0

^a,b^ Slope and intercept represent *a* and *b* in *Y = ax + b* linear model. *Y* means peak area and *x*, concentration.

**Table 3:** Antioxidant activities of spirulina samples.

Items	Samples							
	AP-1	C1	C2	C3	C4	C5	C6	C7
Antioxidant activity of carotenoid extracts								
Total phenolics (μmol GAE/g)	1.3 ± 0.1 ^d^	2.3 ± 0.4 ^c^	1.5 ± 0.2 ^d^	4.6 ± 0.6 ^b^	2.9 ± 0.5 ^c^	6.4 ± 0.6 ^a^	2.9 ± 0.4 ^c^	2.5 ± 0.0 ^c^
Total flavonoids (μmol QE/g)	26.6 ± 2.4 ^a^	22.8 ± 3.4 ^a^	24.0 ± 1.0 ^a^	15.6 ± 0.9 ^b^	15.0 ± 2.4 ^b^	12.9 ± 0.4 ^c^	18.5 ± 1.9 ^b^	24.0 ± 2.9 ^a^
DPPH (μmol TE/g)	18.5 ± 0.5 ^a^	8.4 ± 1.3 ^c^	10.3 ± 1.1 ^b^	6.1 ± 0.8 ^d^	5.8 ± 1.2 ^d^	5.2 ± 0.8 ^d^	7.3 ± 0.4 ^c^	6.4 ± 0.8 ^d^
ABTS (μmol TE/g)	33.7 ± 5.0 ^a^	18.7 ± 3.9 ^b^	23.6 ± 1.6 ^b^	24.8 ± 4.2 ^b^	21.4 ± 3.6 ^b^	18.1 ± 2.0 ^b^	19.3 ± 3.1 ^b^	19.3 ± 3.2 ^b^
Antioxidant activity of C-PC extracts								
Total phenolics (μmol GAE/g)	82.1 ± 4.8 ^a^	66.2 ± 4.5 ^b,c^	70.6 ± 2.6 ^b^	47.0 ± 5.4 ^c^	53.9 ± 8.8 ^c^	62.4 ± 4.8 ^b,c^	64.3 ± 6.2 ^b,c^	47.6 ± 0.8 ^c^
Total flavonoids (μmol QE/g)	ND	ND	ND	ND	ND	ND	ND	ND
DPPH (μmol TE/g)	18.7 ± 0.2 ^a^	14.8 ± 1.4 ^b^	19.1 ± 1.5 ^a^	18.1 ± 1.6 ^a^	15.5 ± 2.3 ^b^	16.2 ± 1.2 ^b^	16.5 ± 1.1 ^b^	14.4 ± 0.9 ^b^
ABTS (μmol TE/g)	108.3 ± 10.2 ^a^	66.6 ± 3.3 ^c^	83.4 ± 5.1 ^b^	42.7 ± 4.4 ^e^	49.4 ± 1.5 ^d^	51.7 ± 2.7 ^d^	47.3 ± 6.1 ^d,e^	42.5 ± 6.2 ^e^

GAE, gallic acid equivalent; QE, quercetin equivalent; TE, Trolox equivalent. Data are expressed as the mean (the average value for dry weight) and SD (the standard deviation value) of three independent experiments. Different letters in the same row mean significantly different (*p* < 0.05). ND, not detected.

**Table 4:** Pearson’s correlation coefficients of Hunter values, pigment contents and antioxidant activities.

Traits	TC	CA	PC	TP-C	TF-C	DPPH-C	ABTS-C	TP-P	DPPH-P	ABTS-P
L*	–0.12	–0.11	–0.28	–0.29	0.25	–0.01	–0.12	–0.09	–0.24	–0.25
a*	0.58 **	0.39	0.50 *	–0.12	0.13	0.42 *	0.22	0.66 **	0.39	0.68 **
b*	–0.32	–0.41 *	–0.54 **	–0.10	–0.15	–0.26	–0.24	–0.19	–0.21	–0.42 *
TC		0.92 **	0.86 **	–0.63 **	0.66 **	0.97 **	0.71 **	0.83 **	0.50 *	0.95 **
CA			0.94 **	–0.45 *	0.55 **	0.94 **	0.80 **	0.68 **	0.50 *	0.86 **
PC				–0.30	0.42 *	0.87 **	0.74 **	0.71 **	0.53 **	0.86 **
TP-C					–0.82 **	–0.63 **	–0.37	–0.40	–0.12	–0.57 **
TF-C						0.67 **	0.31	0.44 *	0.07	0.60 **
DPPH-C							0.77 **	0.80 **	0.52 **	0.90 **
ABTS-C								0.43 *	0.53 **	0.65 **
TP-P									0.55 **	0.83 **
DPPH-P										0.49 *

TC, total carotenoid content; CA, chlorophyll a content; PC, phycocyanin content; TP-C, total phenolic content in carotenoid extract; TF-C, total flavonoid content in carotenoid extract; DPPH-C, antioxidant activity of carotenoid extract on DPPH assay; ABTS-C, antioxidant activity of carotenoid extract on ABTS assay; TP-P, total phenolic content in C-PC extract; DPPH-P, antioxidant activity of C-PC extract on DPPH assay; ABTS-P, antioxidant activity of C-PC extract on ABTS assay. * Significant at *p* < 0.05. ** Significant at *p* < 0.01.
